# Supplementary material for: Targeting Oral Squamous Cell Carcinoma with Combined Polo-Like-Kinase-1 Inhibitors and γ-Radiation Therapy
Source: Biomedicines. 2024 Feb 23;12(3):503. doi: 10.3390/biomedicines12030503 (PMC10968374; doi:10.3390/biomedicines12030503)
Supplement: Supplementary file 1 [file biomedicines-12-00503-s001.zip › biomedicines-2802334-supplementary/biomedicines-2802334-Supplementary_tables.docx]

**Table S1.** High-throughput Drug Screen on CAL27 cells.

| **Class of inhibitor** | **Inhibitor name** | **IC_50_ range in CAL27** |
| --- | --- | --- |
| 5α-reductase inhibitor | Dutasteride (Avodart) | >10uM |
| Abl/Src inhibitor | Bosutinib (SKI-606) | 1-10uM (4) |
| Adenosine Receptor Agonist | CGS 21680 | >10uM |
| Adenosine Receptor Antagonist | CVT-6883 | >10uM |
| AHR Antagonist | SR1 | 1-10uM (9) |
| Akt Inhibitor | MK-2206 | >10uM |
|  | GSK690693 | 1-10uM (7.5) |
| AMPK Activator | A-769662 | >10uM |
| Androgen Receptor Inhibitor | Bicalutamide (Casodex) | >10uM |
| Antihistamine | Dimebolin Hydrochloride | >10uM |
| Angiotensin Receptor Antagonist | Eprosartan Mesylate | >10uM |
| Aurora Kinase Inhibitor | ZM 447439 | >10uM |
|  | VX-680 | <0.01uM |
|  | AZD1152 | 0.01-0.1uM (0.07) |
| Bcl-2 Inhibitor | ABT-263 | 1-10uM (4) |
|  | ABT-737 | 1-10uM (9) |
| Bcr-Abl Inhibitor | Nilotinib | 1-10uM (8) |
|  | Ponatinib (AP24534) | 0.1-1uM (0.65) |
| B-Raf Inhibitor | PLX4032 (RG7204) | 10uM |
|  | PLX4720 | 1-10uM (5.5) |
|  | Dabrafenib | >10uM |
| Bruton's TK Inhibitor | PCI-32765 | 0.1-1uM (0.8) |
| Cathepsin Inhibitor | Odanacatib (MK-0822) | >10uM |
| CCR5 Antagonist | Maraviroc (UK-427857) | >10uM |
| CDK Inhibitor | PD-0332991 | 0.1-1uM (0.25) |
| Checkpoint Kinase Inhibitor | AZD7762 | 0.1-1uM (0.5) |
|  | SCH900776 | 1-10uM (5.5) |
| c-Met Inhibitor | PF-2341066 | 1uM |
|  | ARQ 197 (Tivantinib) | 0.1-1uM (0.3) |
|  | BMS-777607 | 10uM |
|  | EMD1214063 | 1-10uM (8.5) |
|  | PF-04217903 | >10uM |
|  | [VEGFR2] Foretinib | 0.1-1uM (0.5) |
| COX-2 Inhibitor | Rofecoxib (Vioxx) | >10uM |
| CRTH2-R Antagonist | Ramatroban (Bay u3405) | >10uM |
|  | TM30089 | >10uM |
| EGF-R Inhibitor | [HER1/2] Lapatinib | >10uM |
|  | Erlotinib, Hydrochloride | 1-10uM (5.5) |
|  | [HDAC/HER2] CUDC-101 | 0.01-0.1uM (0.085) |
|  | WZ4002 | 1-10uM (9) |
| ErbB-R Inhibitor | Canertinib (CI-1033) | 1-10uM (3) |
|  | Dacomitinib | 0.1-1uM (0.3) |
| ERK2 Inhibitor | VX-11e | 0.1-1uM (0.45) |
| FGFR Inhibitor | AZD4547 | 1-10uM (9) |
|  | NVP-BGJ398 | 1-10uM (8) |
| FLT3 Inhibitor | AC220 (Quizartinib) | >10uM |
| HDAC Inhibitor | LBH-589 (Panobinostat) | 0.01-0.1uM (0.03) |
|  | MS-275 | 0.1-1uM (0.8) |
|  | MGCD0103 | 0.1-1uM (0.7) |
|  | Vorinostat (SAHA) | 0.1-1uM (0.8) |
|  | Belinostat (PXD101) | 0.1-1uM (0.55) |
|  | [EGF-R/HER2] CUDC-101 | 0.01-0.1uM (0.085) |
|  | Tubacin | 1-10uM (8.5) |
|  | Tubastatin A | 1-10uM (9.3) |
| Hedgehog Pathway Inhibitor | GDC-0449 | >10uM |
| HER1/2 Inhibitor | [EGF-R] Lapatinib | >10uM |
|  | [HDAC/EGF-R] CUDC-101 | 0.01-0.1uM (0.085) |
| HIV Integrase Inhibitor | Raltegravir | >10uM |
| HMG-CoA Reductase Inhibitor | Atorvastatin Calcium (Lipitor) | 1-10uM (8.5) |
| HSP 90 Inhibitor | 17-DMAG | <0.01uM (0.002866) |
|  | 17-AAG | 0.01uM |
|  | NVP-AUY922 (VER-52296/Novartis) | <0.01uM |
| ICE/Caspase-1 Inhibitor | VX-765 | >10uM |
| IGF-1R Inhibitor | OSI-906 | 1-10uM (8) |
|  | BMS-754807 | 1-10uM (5) |
| Immunodulator | Lenalidomide (CC-5013) | >10uM |
| Immunosuppressant | FTY720, Hydrochloride | 1-10uM (7) |
|  | FK-506 | >10uM |
| JAK Inhibitor | [JAK3] CP-690550 | >10uM |
|  | AZD1480 | 1-10uM (6.5) |
|  | CYT-387 | 1-10uM (3.5) |
|  | INCB018424 (Ruxolitinib) | >10uM |
|  | TG101348 | 1-10uM (6) |
| LTR Antagonist | Montelukast Sodium (Singulair) | >10uM |
| MEK Inhibitor | PD 0325901 | 0.01uM |
|  | RDEA119 | 0.1-1uM (0.4) |
|  | AZD 6244 (ARRY-142886) | 0.1uM |
|  | ARRY-162 | 0.1-1uM (0.2) |
|  | GSK1120212 | <0.01uM |
|  | AS703026 (MSC1936369B) | 0.01-0.1uM (0.05) |
| mTOR Inhibitor | Pp242 | 1-10uM (4) |
|  | Rapamycin (Sirolimus) | 1-10uM (4) |
|  | AZD8055 | 0.1-1uM (0.5) |
|  | INK128 | 0.01-0.1uM (0.075) |
|  | OSI-027 | 1-10uM (3.5) |
|  | [PI3K] PF-04691502 | 0.1-1uM (0.55) |
|  | [PI3K] GDC-0980 | 0.1-1uM (0.5) |
| Multi-Kinase Inhibitor | Regorafenib (BAY 73-4506) | 1-10uM (8.5) |
| NA Synthesis Inhibitor | Capecitabine (Xeloda) | >10uM |
|  | Pemetrexed Disodium (Alimta) | >10uM |
|  | Gemcitabine, HCl (Gemzar) | <0.01uM |
|  | Doxorubicin (Adriamycin) | <0.01uM |
| NPM-ALK Inhibitor | NVP-TAE684 | 1-10uM (6) |
| p38 MPK Inhibitor | VX702 | >10uM |
| p90 RSK Inhibitor | BI-D1870 | 1-10uM (7) |
| PARP Inhibitor | ABT-888 (Veliparib) | >10uM |
|  | AZD 2281 (Olaparib) | 1-10uM (8) |
|  | AG014699 (PF-01367338) | 10uM |
|  | BSI-201 (Iniparib) | >10uM |
|  | MK-4827 | 1uM |
| PDE4 Inhibitor | AN2728 | >10uM |
| PGD2-R Antagonist | Laropiprant | >10uM |
| Phospholipase A2 Inhibitor | Varespladib (LY315920) | >10uM |
| PI3K Inhibitor | TGX-221 | >10uM |
|  | PIK-75, Hydrochloride | <0.01uM (0.01525) |
|  | GDC-0941 | 1-10uM (4) |
|  | CAL-101 | >10uM |
|  | LY294002 | 1-10uM (7.5) |
|  | [mTOR] PF-04691502 | 0.1-1uM (0.55) |
|  | TG100-115 | >10uM |
|  | XL-147 | >10uM |
|  | [mTOR] GDC-0980 | 0.1-1uM (0.5) |
|  | NVP-BKM120 | 0.1-1uM (0.8) |
| **PLK Inhibitor** | **BI 2536** | **<0.01uM** |
|  | **BI 6727 (Volasertib)** | **<0.01uM (0.0009856)** |
|  | **GSK461364** | **0.1-1uM (0.75)** |
| Protease Inhibitor | VX-950 (Telaprevir) | 10uM |
| Proteasome Inhibitor | Bortezomib (Velcade) | <0.01uM |
|  | Carfilzomib (PR-171) | <0.01uM (0.00002632) |
| RTK Inhibitor | BIBW 2992 (Tovok) | 1-10uM (3) |
|  | Vandetanib (Zactima) | 1-10uM (8) |
|  | Vatalanib Dihydrochloride | >10uM |
|  | BMS-599626 | >10uM |
|  | Motesanib (AMG-706) | >10uM |
|  | Tandutinib | >10uM |
| RXR Activator | Bexarotene (Targretin) | 10uM |
| SMO Inhibitor | NVP-LDE225 | >10uM |
| Src Inhibitor | AZD05030 (Saracatinib) | 1-10uM (6) |
|  | [Abl] Bosutinib (SKI-606) | 1-10uM (4) |
|  | Dasatinib | <0.01uM |
| Survivin Suppressant | YM155 | 0.01-0.1uM (0.05) |
| T Cell Activation Inhibitor | Hypothemycin | 1-10uM (3) |
| TK Inhibitor | Imatinib | >10uM |
|  | Gefitinib (Iressa) | >10uM |
|  | Sorafenib | 1-10uM (7) |
|  | Sunitinib | 1-10uM (8) |
|  | Pazopanib (Votrient) | >10uM |
|  | XL-184 (Cabozantinib) | 1-10uM (5.5) |
| Topoisomerase I Inhibitor | SN-38 | <0.01uM (0.008960) |
|  | Topotecan (Hycamtin) | <0.01uM (0.001348) |
| Topoisomerase II Inhibitor | Etoposide | 0.1-1uM (0.8) |
| Tubulin Stabilizer | Docetaxel | <0.01uM |
|  | Paclitaxel (Taxol) | <0.01uM |
| VEGF-R Inhibitor | Axitinib (AG-013736) | 1-10uM (8) |
|  | AV-951 (Tivozanib) | >10uM |
|  | [c-MET] Foretinib | 0.1-1uM (0.5) |

**Table S2.** IC_50_ of two PLK-1 inhibitors on different cell lines.

| **Name of Cell Lines** | **BI2536 IC_50_ (nM)** | **Volasertib IC_50_(nM)** | **Sensitive(S)/ Resistant(R)** |
| --- | --- | --- | --- |
| Hs68 (normal) | Not reached | Not reached | R |
| OKF6/TERT-2 (normal) | Not reached | Not reached | R |
| CAL 27 | 3.81 ± 2.17 | 4.54 ± 1.91 | S |
| UMSCC51 | 5.53 ± 2.40 | 4.73 ± 2.17 | S |
| CAL 33 | 6.06 ± 3.80 | 4.83 ± 2.93 | S |
| UMSCC1 | 4.77 ± 2.55 | 5.66 ± 3.55 | S |
| UMSCC59 | 82.97 ± 15.28 | 22.68 ± 13.75 | S |
| UMSCC29 | 71.66 ± 25.02 | 79.66 ± 17.03 | S |
| UMSCC103 | 122.62± 27.70 | 94.366± 8.17 | S |
| UMSCC7 | Not reached | Not reached | R |
| UMSCC43 | Not reached | Not reached | R |
