# Supplementary material for: Targeting Oral Squamous Cell Carcinoma with Combined Polo-Like-Kinase-1 Inhibitors and γ-Radiation Therapy
Source: Biomedicines. 2024 Feb 23;12(3):503. doi: 10.3390/biomedicines12030503 (PMC10968374; doi:10.3390/biomedicines12030503)

Supplementary Figure S1

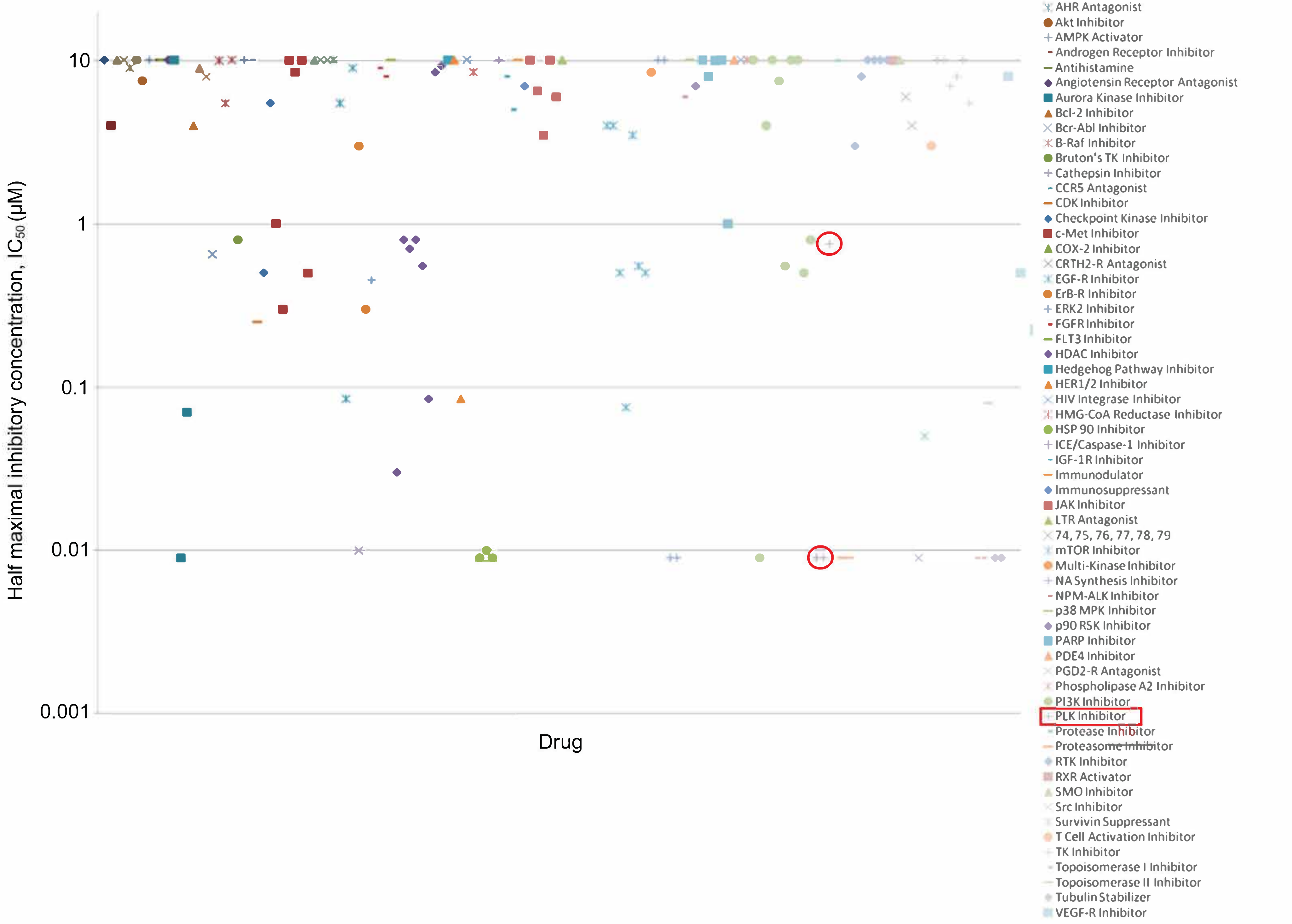

Supplementary
Figure S2

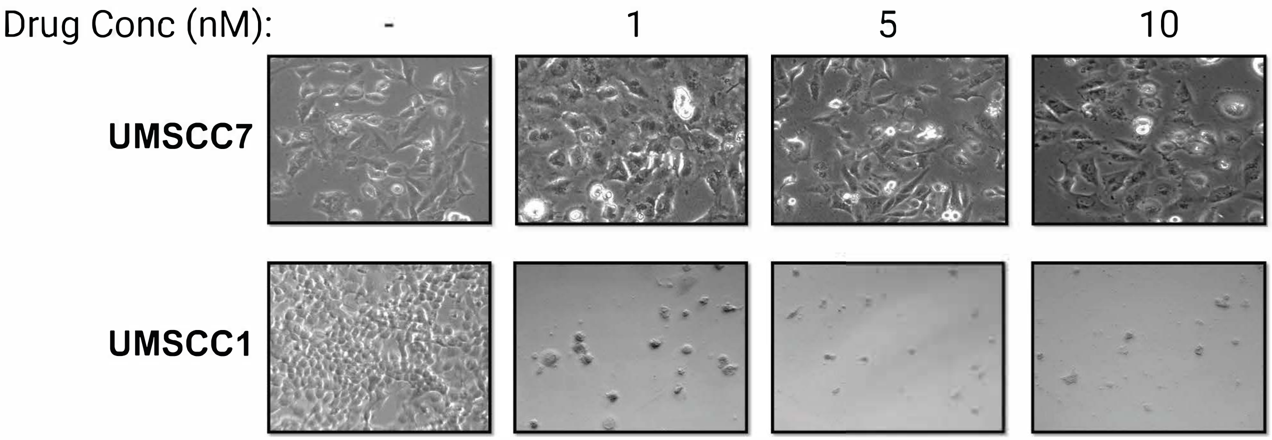

Supplementary
Figure S3

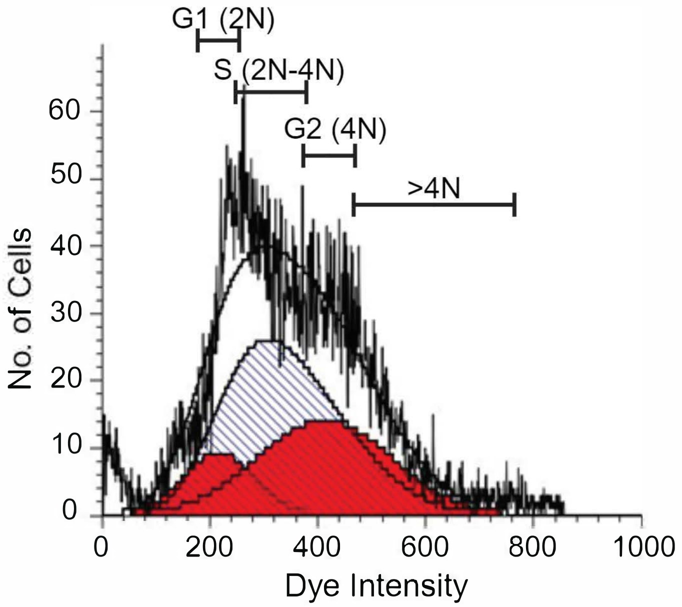

Supplementary Figure S4

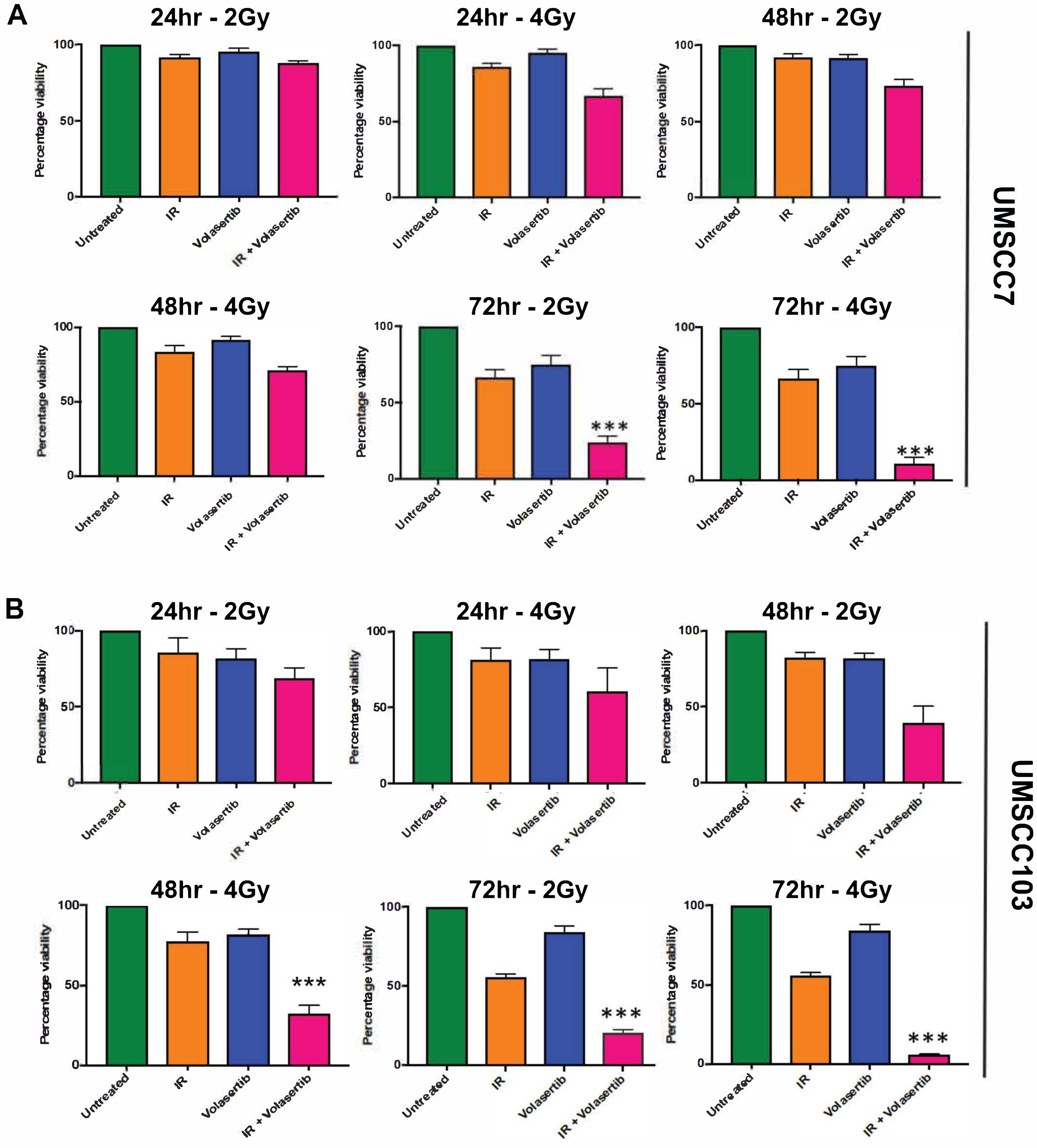

Supplementary Figure S5

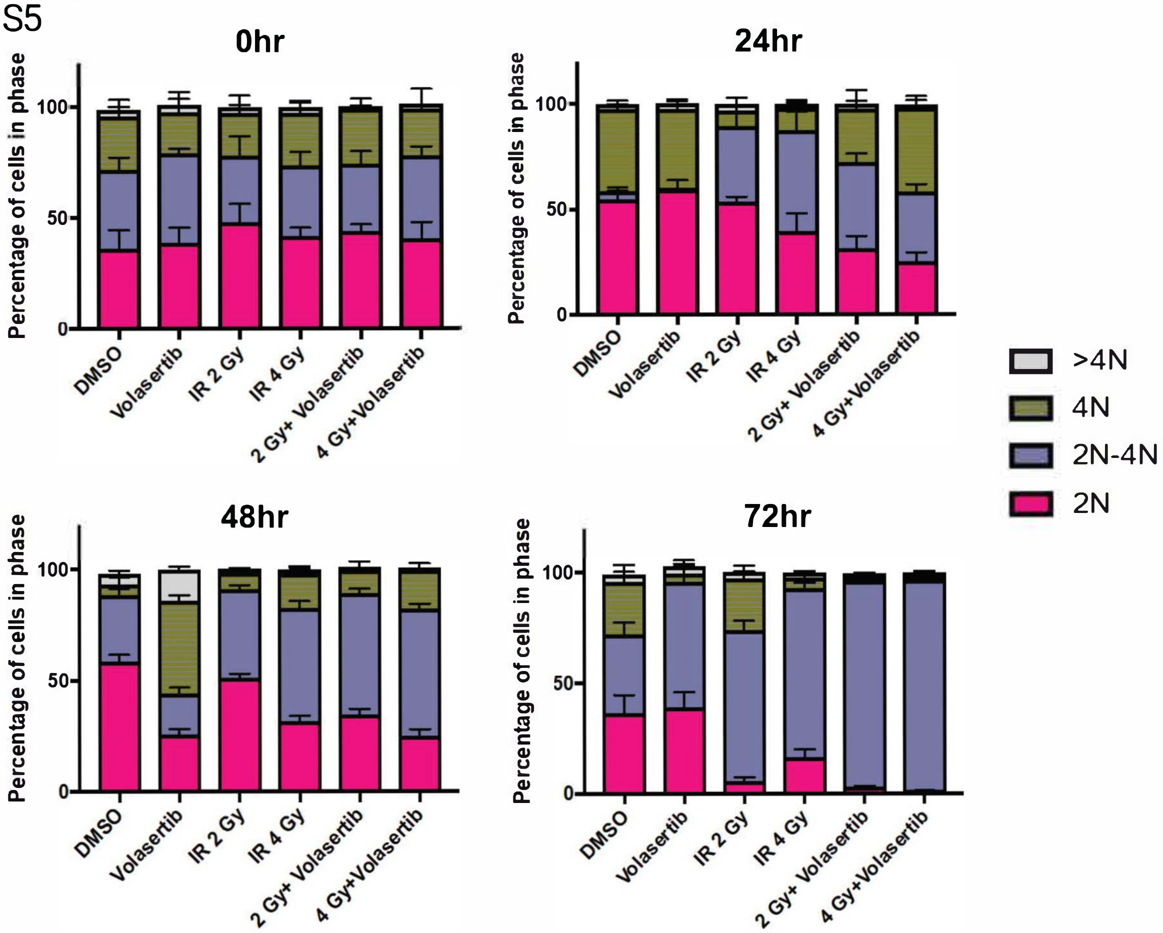

Supplement: Supplementary file 1 [file biomedicines-12-00503-s001.zip › biomedicines-2802334-supplementary/biomedicines-2802334_Supplementary Figures.pdf]
